# Purification and Characterization of Plantaricin ZJ5, a New Bacteriocin Produced by *Lactobacillus plantarum* ZJ5

**DOI:** 10.1371/journal.pone.0105549

**Published:** 2014-08-22

**Authors:** Da-Feng Song, Mu-Yuan Zhu, Qing Gu

**Affiliations:** 1 Key Laboratory for Food Microbial Technology of Zhejiang Province, Department of Biotechnology, Zhejiang Gongshang University, Hangzhou, China; 2 State Key Laboratory of Plant Physiology and Biochemistry, College of Life Sciences, Zhejiang University, Hangzhou, China; University of Kansas Medical Center, United States of America

## Abstract

The aim of this study is to investigate the antimicrobial potential of *Lactobacillus plantarum* ZJ5, a strain isolated from fermented mustard with a broad range of inhibitory activity against both Gram-positive and Gram-negative bacteria. Here we present the peptide plantaricin ZJ5 (PZJ5), which is an extreme pH and heat-stable. However, it can be digested by pepsin and proteinase K. This peptide has strong activity against *Staphylococcus aureus*. PZJ5 has been purified using a multi-step process, including ammonium sulfate precipitation, cation-exchange chromatography, hydrophobic interactions and reverse-phase chromatography. The molecular mass of the peptide was found to be 2572.9 Da using matrix-assisted laser desorption/ionization time-of-flight mass spectrometry (MALDI-TOF MS). The primary structure of this peptide was determined using amino acid sequencing and DNA sequencing, and these analyses revealed that the DNA sequence translated as a 44-residue precursor containing a 22-amino-acid N-terminal extension that was of the double-glycine type. The bacteriocin sequence exhibited no homology with known bacteriocins when compared with those available in the database, indicating that it was a new class IId bacteriocin. PZJ5 from a food-borne strain may be useful as a promising probiotic candidate.

## Introduction

Bacteriocins are ribosomally synthesized peptides that in most cases, exhibit antibacterial activity against bacteria that are closely related to the producing bacteria. Bacteriocins from lactic acid bacteria (LAB) are mostly small, heat-stable, hydrophobic, and cationic peptides [1], [2]. These peptides have attracted significant attention because of their possible applications as non-toxic additives for food preservation and prevention of food spoilage by food-borne pathogenic bacteria [3], [4], [5].

Depending on their structural characteristics, LAB bacteriocins have been classified by Klaenhammer (1993) into three main groups [6]: class I is small (<5 kDa) peptides called lantibiotics, which contains post-translationally modified amino acid residues, such as lanthionine and 3-methyllanthionine; class II is small, heat-stable, nonmodified and membrane-active peptides; and class III is large (>10 kDa) and heat-labile peptides [7]. Most bacteriocins belong to class II, which are divided into four groups, namely class IIa (pediocin-like peptide bacteriocins), IIb (two-peptide bacteriocins), IIc (circular peptide bacteriocins), and IId (nonpediocin linear one peptide bacteriocins).

Nisin is the most typical bacteriocin and is used as a food preservative worldwide [8], [9]. However, the application of nisin is limited because it exhibits activity against Gram-positive bacteria [10]. More than 20 plantaricins have been found, and a number of plantaricins have been purified, such as plantaricin JK [11], plantaricin 1.25 [12], plantaricin NC8 [13], Plantaricin C [14] or plantaricin PASM1 [15]. Some plantaricins, such as plantaricin PASM1 and plantaricin 1.25, show a narrow antibacterial spectrum, exhibiting antibacterial activity against bacteria closely relate to the producer microorganism such as *lactobacillus* strains, similar to many bacteriocins. Plantaricin C, plantaricin JK and plantaricin NC8, like nisin, appear to inhibit some Gram-positive bacteria, such as *Lactobacillus sake*, *Enterococcus faecalis*, and *Bacillus subtilis*, but have no activity against Gram-negative bacteria. Therefore, more broad-spectrum antimicrobial bacteriocins are desired.

We screened LAB strains to obtain new bacteriocins that exhibited antibacterial activity not only against Gram-positive bacteria but also against Gram-negative bacteria. We have isolated more than 150 LAB strains from 13 varieties of fermented mustards obtained from supermarkets in China. A LAB strain known as ZJ5, which produced bacteriocins, was obtained. Here, the identification, purification, and characterization bacteriocin of the strain ZJ5 is described. In addition, the gene encoding the bacteriocin was cloned and sequenced.

## Materials and Methods

### Bacterial Strains and Media

The bacterial strains that served as indicator strains are listed in Table 1. All LAB strains were grown in deMan Rogosa Sharpe (MRS) broth at 30°C without agitation. The other bacteria were grown in LB or YPD broth at 37°C with agitation. The growth of strain ZJ5 was measured turbid metrically at OD600. At least two separate experiments were conducted for each test organism. All media components were purchased from Merck, Germany. Other biochemicals were procured from Sigma-Aldrich, USA.

**Table 1:** Antimicrobial activity of PZJ5 against indicator strains.

Indicator species	Strain^a^	MIC (µM)^b^
*Micrococcus luteus*	CGMCC 1.193	0.153±0.008
*Staphylococcus aureus*	CGMCC 1.879	0.132±0.005
*Staphylococcus aureus*	CGMCC 1.128	0.218±0.013
*Staphylococcus aureus*	ATCC 6538P	0.178±0.025
*Staphylococcus aureus*	CGMCC 1.2386	0.210±0.024
*Lactobacillus plantarum*	CGMCC 1.551	0.722±0.075^c^
*Lactobacillus plantarum*	CGMCC 1.124	0.242±0.014
*Lactobacillus plantarum*	CGMCC 1.11	0.885±0.079
*Lactobacillus plantarum*	CGMCC 1.511	1.352±0.136
*Lactobacillus plantarum*	CGMCC 1.556	0.682±0.018
*Lactococcus lactis*	ATCC 15577	1.225±0.129
*Bacillus subtilis*	CGMCC 1.1627	0.121±0.006
*Enterococcus faecalis*	CGMCC 1.125	0.146±0.027
*Shigella. flexneri*	CGMCC 1.1868	0.135±0.015
*Listeria monocytogenes*	ATCC 7648	0.112±0.009
*Pseudomonas aeruginosa*	CGMCC 1.647	0.144±0.010
*Shigella dysenteriae*	ATCC 9753	0.185±0.011
*Escherichia coli*	JM109	0.135±0.015
*Escherichia coli*	CGMCC 1.1580	0.235±0.038
*Salmonella spp.*	CGMCC 1.1552	0.136±0.012
*Pseudomonas putida*	CGMCC 1.645	0.075±0.003
*Rhodotorula rubra*	CGMCC 2.1034	NA^d^^,^ e
*Saccharomyces cerevisiae*	CGMCC 2.1643	NA^d^

a ATCC, American Type Culture Collection, Rockville, MD; CGMCC, China General Microbiological Culture Collection Center, Peking, China.

b MIC was determined by the agar-well diffusion method [18].

c MRS medium was used instead of LB medium.

d YPD medium was used instead of LB medium.

e NA, no activity.

### The Detection of Inhibitors and Taxonomic Identification

Fermented mustards (10 g) were homogenized in 90 mL of saline solution and then planted in serial dilutions in MRS media. Plates were aerobically incubated at 30°C for 48 h. Several colonies were picked at random for bacteriocin screening and replicated onto four sets of agar plates. After 8 hours of culture, three sets of agar plates were overlaid with 10 mL of overnight culture from the indicator strains *Escherichia coli*, *Staphylococcus aureus*, and *Micrococcus luteus*. After incubation at 37°C, all plates were examined for inhibition zones around individual colonies.

The ZJ5 strain was selected because of its strong antibacterial activity against three indicator strains, and it was subsequently subjected to phenotypic and genotypic identification. This strain was identified based on its cell morphology as observed by microscopy, gram staining, and catalase reaction. Total DNA from the bacteriocin-producing strain was obtained by the alkaline lysis method [16]. The 16S rRNA gene was amplified and sequenced [17].

### Purification of Bacteriocin

Purification was performed by a multi-step protocol [13], [15]. Three liters of culture supernatant from the ZJ5 strain grown in MRS at 30°C for 24 h at a pH of 4.0 was subjected to ammonium sulfate precipitation. Supernatants of *L. plantarum* ZJ5 cultures were precipitated with ammonium sulfate at 80% (wt/vol). The precipitate, collected by centrifugation, was dissolved in 50 mM sodium phosphate buffer, pH 6.0 (buffer A) and desalted by dialysis (100 Da cut-off membrane, Sangon, China). The bacteriocin was then loaded onto an SP-Sepharose Fast Flow cation-exchange column (GE Healthcare, Sweden) in the ÄKTA purifier system (GE Healthcare, Sweden). The column was equilibrated with buffer A at a flow rate of 1 ml/min, and the bacteriocin was eluted using a NaCl gradient (0% to 100% of 1 M) in buffer A in 1 ml fractions. The protein concentration was monitored at 215 nm, and activity was determined in terms of AU/ml.

Then the eluted active fraction was subjected to a Resource ETH (GE Healthcare, Sweden) equilibrated with 1 M NaCl in 10 mM sodium phosphate buffer at pH 5.0 (buffer B). The active fraction was then eluted with a NaCl gradient (100% to 0% of 1 M) in buffer B in 1 ml fractions.

For further purification, the active eluted solution was applied to a Zorbax SB-C18 column (Agilent, USA) in an LC-2000 Plus high-performance liquid chromatography (HPLC) system (Jasco, Tokyo, Japan). Then the active fraction was eluted with a gradient (0% to 100%) of Milli-Q water-acetonitrile containing 0.06% trifluoroacetic acid at 1 ml/min.

### Bacteriocin Activity Assay

The agar-well diffusion method [18] was utilized to detect the antibacterial spectrum of the ZJ5 fractions, which acquired during the purification process. The samples were adjusted to pH 5.0. As an indicator strain, *Escherichia coli* was inoculated in LB agar (ca. 10^5^ CFU/mL), and wells (8 mm diam) were punched in the plate. The wells were filled with 100 µL of samples, and the plates were incubated overnight at 30°C. The diameter of the inhibition zones (mm) around the wells was measured. The MIC is defined as the minimum PZJ5 concentration that yielded a clear zone of growth inhibition in the indicator lawn. All activity measurements were conducted at least three times.

### Polyacrylamide Gel Electrophoresis

To determine the purity and molecular weight, the purified peptide was subjected to tricine-sodium dodecyl sulphate-polyacrylamide gel electrophoresis (tricine-SDS-PAGE). After electrophoresis, the gel was cut into two parts, one of which was stained while the other was fixed in 20% (v/v) isopropanol, 10% (v/v) acetic acid for 1.5 h, rinsed in 0.5% Tween 80 for 16 h and subsequently rinsed in ddH_2_O overnight. The gel was then overlaid with soft agar containing the indicator strain (10^6^ colony-forming units) and was incubated overnight at 37°C.

### Stability Against pH, Heat, and Enzyme Conditions

To determine the sensitivity of the purified peptide to pH, plantaricin ZJ5 was re-suspended in different buffer solutions with pH ranging from 2.0 to 8.0 (50 mM HCl-KCl: pH 2.0, and 4.0; 50 mM phosphate buffer: pH 6.0 and 7.0; and 50 mM Tris-Cl: pH 8.0) and incubated for 2 h at 37°C. Thermo stability was determined at different temperatures in experiments where the protein samples were boiled (100°C in a water bath for 30 min) and autoclaved (121°C at 15 psi for 15 min) prior to assaying for activity [15].

Plantaricin ZJ5 was treated with the following enzymes at 1 mg/mL: trypsin (8.0), α-chymotrypsin (8.0), proteinase K (7.5), pepsin (2.0), papain (7.0), lipase (9.0), and α-amylase (6.0) (Sigma-Aldrich, USA). After incubation at 37°C for 2 h, enzyme activity was terminated by heating at 100°C for 5 min. Then residual plantaricin activity was determined using the agar-well diffusion method described above using *Escherichia coli* as an indicator strain. The area of inhibition was calculated from the diameter of the inhibition zones, and the decrease ratio was displayed as a percentage. Untreated peptide samples were taken as respective controls [19].

### N-terminal Amino Acid Sequencing and Molecular Mass Spectrometry

The molecular mass was characterized by using matrix-assisted laser desorption/ionization time-of-flight mass spectrometry (MALDI TOF-Target) (Bruker Daltonics, USA). The HPLC-purified peptide plantaricin ZJ5 was analyzed via automated Edman degradation using a PPSQ-31A Protein Sequencer (Shimadzu Corporation, Japan).

### DNA Sequence Analysis

DNA polymerase was purchased from TaKaRa Bio, Tokyo, Japan. Chromosomal DNA from the ZJ5 strain was obtained by the alkaline lysis method [16] and used as a DNA template for PCR amplification. Table 2 listed all the primers used in this study. The degenerate primers AGF1 and AGR1, which were designed from the amino acid sequences obtained, were used to amplify a genomic DNA fragment containing a portion of *PZJ5*. To amplify the upstream and downstream areas of the complete sequence, thermal asymmetric interlace PCR (Tail-PCR) was performed as described previously [20], [21]. Chromosomal DNA was used as a template for PCR with a structural gene-specific primer (LB-TR1) and random primer (AD1-AD5). The second (with primers LB-TR2 and AD1-AD5) and the third (with primers LB-TR3 and AD1-AD5) PCR were performed as described above. The products were purified by a quick PCR purification kit (Sangon, China). Then they were sequenced. The DNA sequence of the downstream region was analyzed using random primers and the following specific primers: RB-TR1, RB-TR2, and RB-TR3. Based on the sequences obtained from the Tail-PCR products, the new specific primers PZJ5-F and PZJ5-R were synthesized and used to confirm the entire gene sequence using the procedures described above.

**Table 2:** Oligonucleotide primers used to obtain the PZJ5 gene structure.

Primers	Sequences (5′-3′)^a^
AGF1	AARACNAARCARCARTT
AGR1	GTRTANCCRAANACYTT
LB-TR1	GTATAACCAAATACTT
LB-TR2	GTGTTTGTGCTTTGAT
LB-TR3	TGATTAAAAATTGTTG
RB-TR1	TTTAATCAAAGCACAA
RB-TR2	ATCAAAGCACAAACAC
RB-TR3	AAGTATTTGGTTATAC
AD Primers1	NTCGASTWTSGWGTT
AD Primers2	NGTCGASWGANAWGAA
AD Primers3	WGTGNAGWANCANAGA
AD Primers4	TGWGNAGSANCASAGA
AD Primers5	AGWGNAGWANCAWAGG
PZJ5-F	AGATTCCAGGCAATG
PZJ5-R	GGAATAAATCAGTTA

In the degenerate primers, N, R, Y, and D indicate A/T/G/C, A/G, T/C, and A/T/G, respectively.

### Analysis of DNA and Amino Acid Sequences

The DNA and amino acid sequences obtained were analyzed using DNAStar software, version 5.0 (DNASTAR, Madison, USA). Database searches were performed using BLAST (NCBI; http://www.ncbi.nlm.nih.gov/BLAST/).

### Nucleotide Sequence Accession Numbers

The sequences of the 16S rRNA gene from the ZJ5 strain were submitted to the GenBank database, and its accession number is KF032707. The nucleotide sequence containing the bacteriocin structural gene presented in this paper has been assigned the accession number KF032708.

## Results

### Identification of Bacteriocin-producing Strain ZJ5

The production of the ZJ5 strain showed strong bacteriocin activity against *E. coli*. The strain was a Gram-positive and catalase-negative bacillus that did not produce gas. The 16S rRNA sequence from ZJ5 had a similarity value of more than 99.9% with *L. plantarum* JDM1 (GenBank accession No. NC012984). The carbohydrate fermentation pattern of the ZJ5 strain was also in agreement with that of other *L. plantarum* strains (data not shown). Therefore, ZJ5 strain could be classified as a member of the *L. plantarum* genus. The strain was designated *L. plantarum* ZJ5, and the bacteriocin produced by this strain was named plantaricin ZJ5 (PZJ5).

### Purification of Plantaricin ZJ5

**Figure 1:**
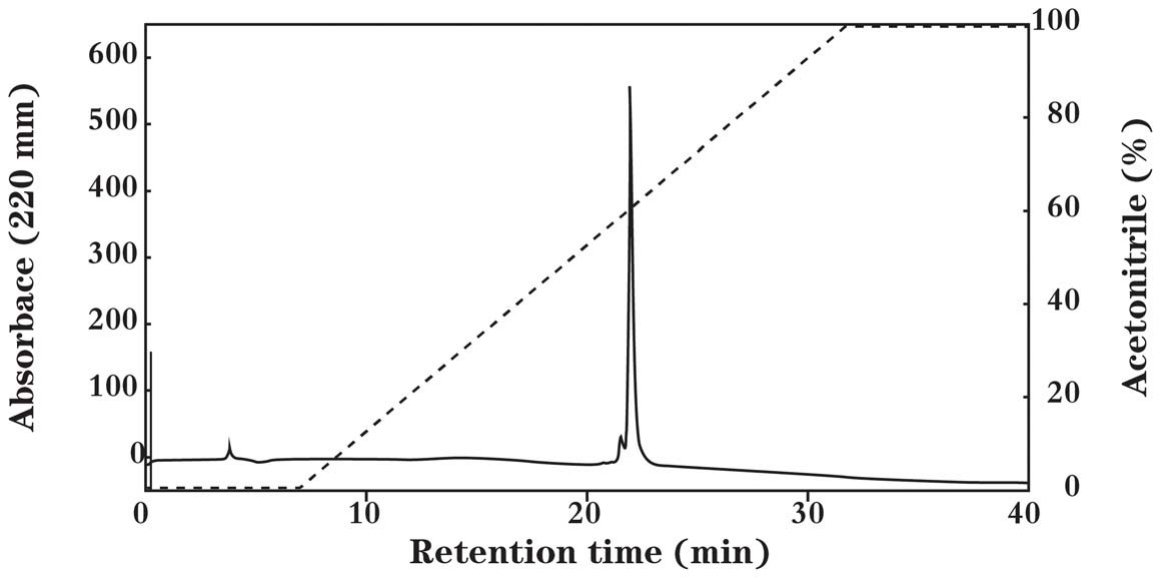
Reverse-phase HPLC analysis of plantaricin ZJ5. Elution was performed with a 0–100% linear gradient of acetonitrile and water containing 0.06% TFA.

**Figure 2:**
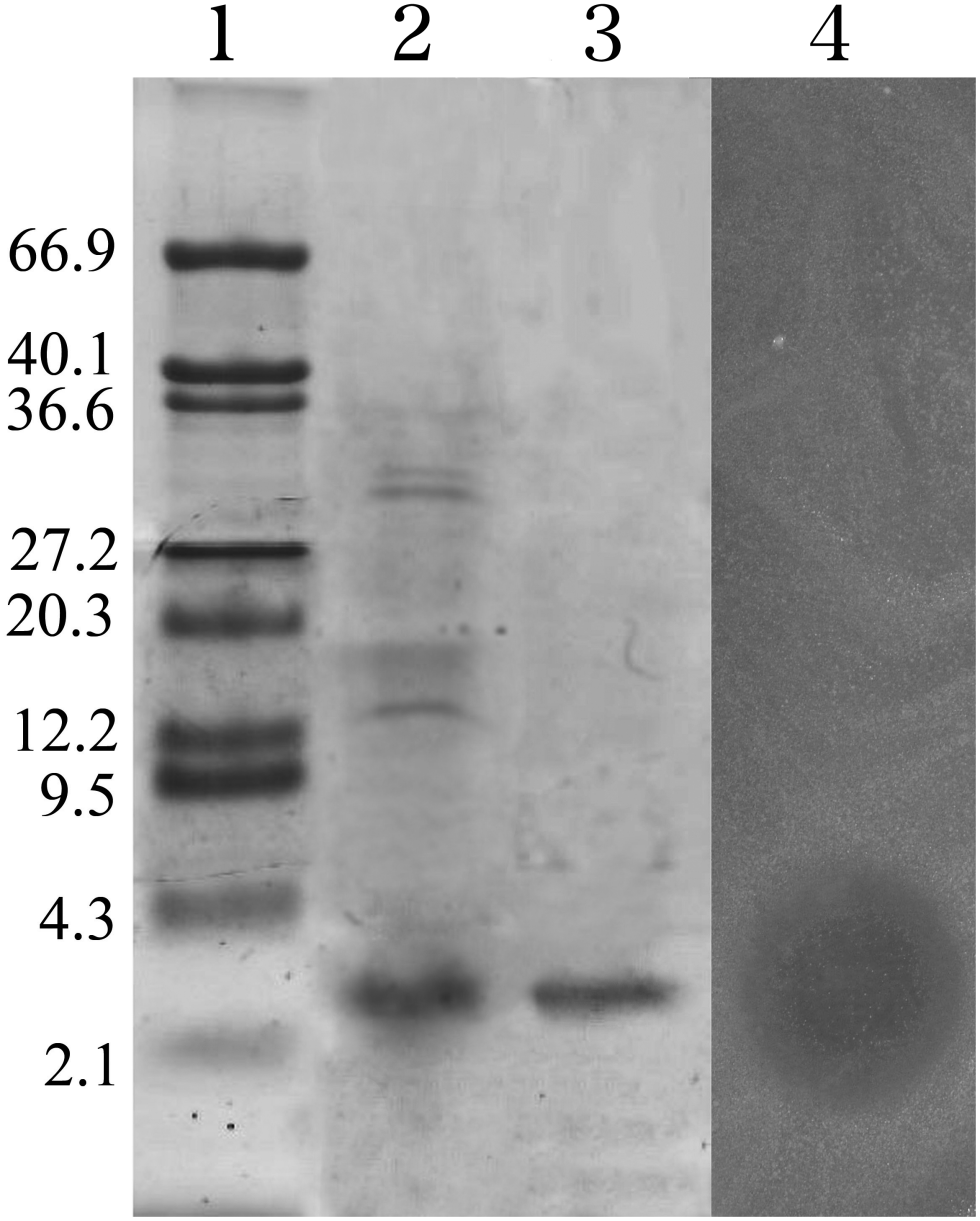
Tricine-Sodium dodecyl sulphate-polyacrylamide gel electrophoresis (tricine-SDS-PAGE) of isolated plantaricin ZJ5. Lane 1, molecular mass marker with the corresponding value in kDa on the left (Tiandz, China); lane 2, PZJ5 precipitated by ammonium sulfate; lane 3, purified PZJ5; lane 4, gel overlaid with indicator strain *Staphylococcus aureus*.

PZJ5 was purified from the culture supernatant by ammonium sulfate precipitation and a two-step procedure, including cation-exchange and hydrophobic-interaction chromatography (Table 3). The active eluted solution obtained using hydrophobic-interaction chromatography was then subjected to reverse-phase HPLC. It produced one peak, which was eluted at an approximately 60% acetonitrile concentration. The active fraction was subjected again to RP-HPLC (Figure 1). The final yield of the peptide obtained in these purification steps was 1.7% from 3 L of the ZJ5 culture supernatant; the details are summarized in Table 3. PZJ5 was analyzed using Tricine-SDS-PAGE. A single protein of ca. 3 kDa was detected and its activity was confirmed by a gel antimicrobial assay (Figure 2).

**Table 3:** Summary of purification index of plantaricin ZJ5 produced by *L. Plantarum* ZJ5.

Purification stage	Volume (ml)	Total protein (mg)	Total activity (AU)	Specific activity^a^ (AU/mg)	Purification fold	Yield (%)
Culture supernatant	3,000	8,445	640,000	75.78	1.0	100
Ammonium sulfate precipitation	200	504.5	160,000	317.14	4.2	25
Cation-exchange chromatography	30	39.21	48,000	1,224.17	16.1	7.5
hydrophobic-interaction chromatography	10	8.76	20,000	2,283.11	30.1	3.1
C18 reverse-phase HPLC	5	1.04	11,000	10,576.92	139.5	1.7

a The specific activity is the ratio between the total activity and total protein

### Characterization of PZJ5

The inhibitory spectrum of PZJ5 is shown in Table 1. The plantaricin ZJ5 spectrum differed from the spectra of previously reported plantaricins, exerting high activity against a broad range of both Gram-positive and Gram-negative bacteria, with particularly strong activity being observed against *Lactobacillus plantarum*, *Listeria monocytogenes*, *Bacillus subtilis*, *Micrococcus luteus*, *Pseudomonas putida*, and *Escherichia coli* but no activity against *Rhodotorula rubra* and *Saccharomyces cerevisiae*. 100% stability was recorded in a wide pH range from 2.0 to 6.0. The activity was not recorded at neutral and alkaline pH, but was regained when the pH was reverted to 5.0 (Table S1). Incubation at different temperatures also led to no losses in activity. 100% activity was recorded even after boiling and autoclaving. When a purified preparation was treated with different hydrolytic enzymes, the inhibitory action was significantly reduced by the proteolytic enzymes. The inhibitory action was completely abolished by pepsin and proteinase K (62%) but not affected after treatment with lipase and α-amylase (Table 4).

**Table 4:** Peptidase sensitivity of plantaricin ZJ5.

Peptidase	Diam (mm)^a^
Control (no peptidase)	25
trypsin	25
Cation-exchange chromatography	25
proteinase K	15
pepsin	0
α-chymotrypsin	25
papain	25
lipase	25
α-amylase	25

a Enzyme treatments were performed at 37°C for 2 h, and *Escherichia coli* was used as the indicator strain. Diameters were based on the clear zones of inhibition.

### Molecular Mass Analysis and Amino Acid Sequencing of Plantaricin ZJ5

**Figure 3:**
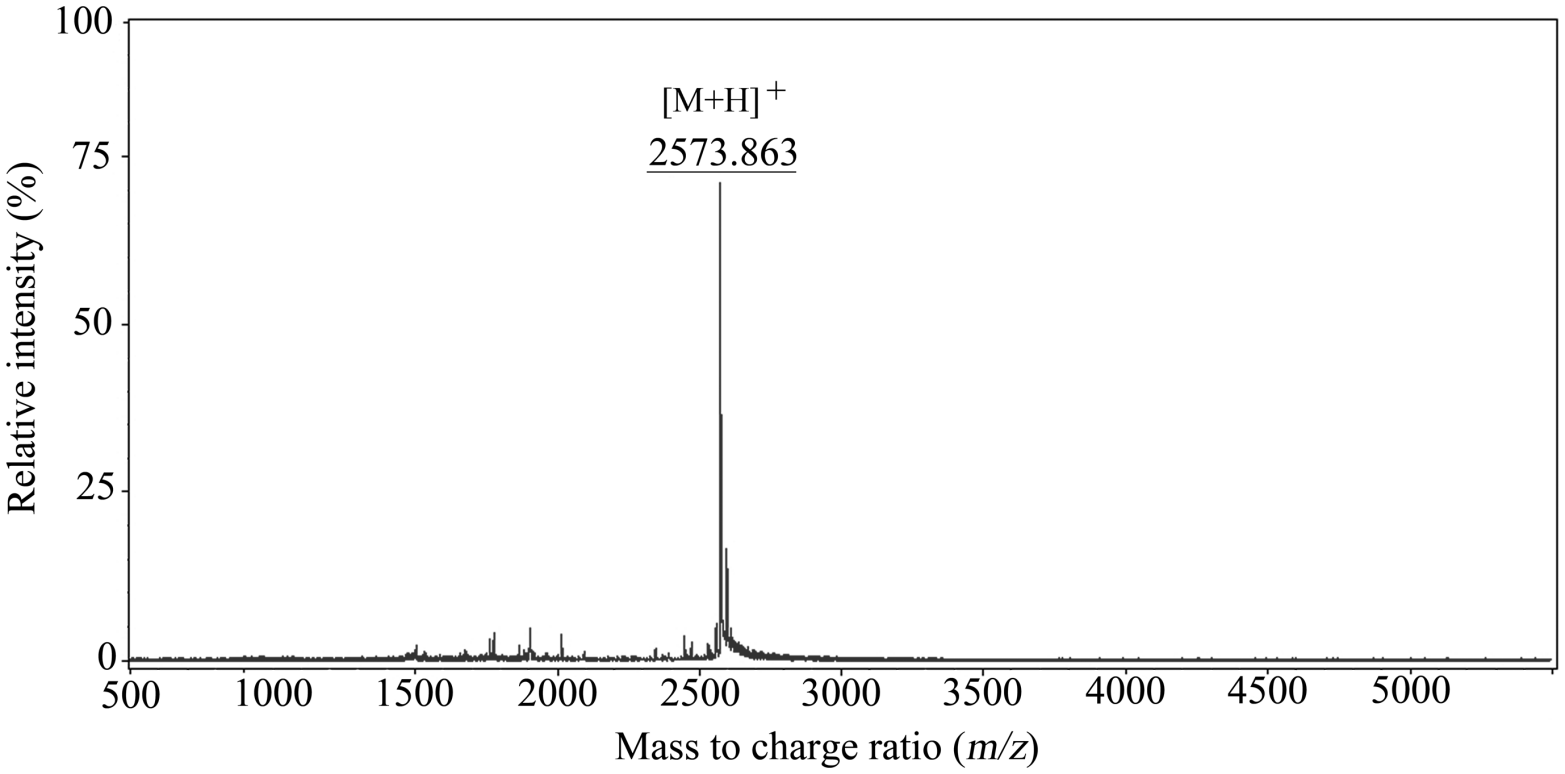
MALDI-TOF mass spectrometry of purified plantaricin ZJ5. A *m/z* 2573.863 monoisotopic peak ([M+H]^+^) is evident.

The molecular mass of the purified peptide was confirmed by MALDI-TOF/MS analysis, and the molecular mass of PZJ5 was determined to be 2572.9 Da (m/z = 2573.9) (Fig. 3). N-terminal amino acid sequence analyses of PZJ5 were performed using automated Edman degradation, and 22 amino acids were determined to be N′-KTKQQFLIKAQTQLFKVFGYTL. The unimpaired Edman degradation sequencing suggests that the bacteriocin belongs to a linear, class II bacteriocin. It's estimated that the isoelectric point was 10.18, and its overall charge at pH 7.0 was 4. The amino acid sequence of plantaricin ZJ5 peptide was compared with NCBI Genbank database and Bactibase based on protein-protein homology (Blastp), indicating that the sequenced residues didn't have apparent homology with other known bacteriocins or proteins.

### Analysis of the Gene Encoding plantaricin ZJ5

**Figure 4:**
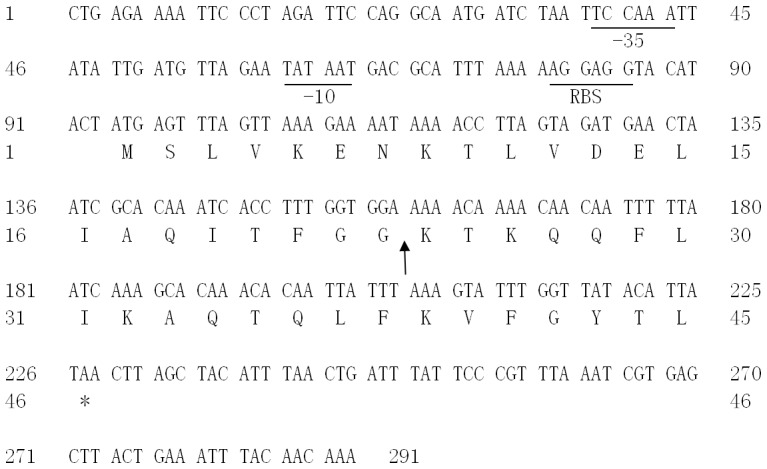
Nucleotide and deduced amino acid sequences in the region containing pzj5. The putative −35 and −10 (Pribnow box) promoter sequences and ribosome binding site (RBS) are underlined. The vertical arrow indicates the Gly-Gly cleavage site of the presequence. The stop codon is indicated with an asterisk.

Degenerate primers were based on the amino acid sequence obtained in the manner described above. When PCR was performed with total DNA from *L. plantarum* ZJ5 and the degenerate primers, a 63 bp fragment was produced. The DNA sequence was used to design specific nests and random primers. These were then used for Tail-PCR and to obtain adjacent sequences. The DNA sequence of the structural gene encoding plantaricin ZJ5 was obtained in this way (Figure 4). Sequence analysis revealed a ribosome binding site, a possible leader peptide sequence, and a terminator sequence. This bacteriocin was found to be translated as a 44 amino acid pre-bacteriocin that was then processed to produce mature plantaricin ZJ5, which consists of 22 amino acids.

A double-glycine leader peptide is used to synthesize plantaricin ZJ5. This leader peptide shares 62% homology with plantaricin ASM1, a plantaricin produced by *Lactobacillus plantarum* A-1 (data not shown).

## Discussion

In this study, we describe the purification and characterization of a new bacteriocin (PZJ5) produced by an environmental isolate strain LAB *L. Plantarum* ZJ5. Plantaricin ZJ5 shows stability under heating and acidic conditions (pH2.0–6.0). However, the peptide demonstrated reduced activity under alkaline conditions. Being proteinaceous in nature, the complete inactivation or significant reduction in the antimicrobial properties of purified plantaricin was observed after treatment with pepsin and proteinase K, but the same was not observed with lipase or α-amylase treatment. Most plantaricins appear to inhibit some Gram-positive bacteria, exhibiting antibacterial activity against bacteria closely related to the producer microorganism and have no activities against Gram-negative bacteria. Plantaricin ZJ5 not only has high activity against Gram-positive bacteria, but also has strong activity against Gram-negative bacteria, such as *Escherichia coli* and *Salmonella spp*. PZJ5 exhibited activity over a wide range of pH, heat, and bactericidal antimicrobial spectrums, signifying that plantaricin ZJ5 could preserve its structure and bactericidal functions even under extreme conditions, which is an important property in view of its potential use as a biopreservative in foods.

Plantaricin ZJ5 was purified from the culture supernatant of *L. Plantarum* ZJ5 using a four-step purification procedure. Because bacteriocin peptides share many common properties, a purification strategy used by several groups could be applied, such that for plantaricin S [22] and NC8 [13]. Plantaricin ZJ5 is cationic and hydrophobic, which was demonstrated by cation-exchange chromatography, hydrophobic-interaction chromatography and RP-FPLC. Both properties are in agreement with earlier reports on bacteriocins [13], [23], [24], [25].

Edman degradation analysis, degenerate PCR and Tail-PCR were performed to obtain the gene structure of plantaricin ZJ5 (Figure 4). Comparing the amino acid sequence of the purified plantaricin ZJ5 peptide with other bacteriocins by using Blastp in NCBI showed no apparent homology with other known bacteriocins or proteins. This result shows apparently a lack of similarity and also suggests that plantaricin ZJ5 is a novel bacteriocin. The lack of unusual amino acids in the entire amino acid sequence of plantaricin ZJ5, heat resistance and low molecular mass indicates that plantaricin ZJ5 belongs to the group of class IId bacteriocins [26], [27].

The DNA sequence of the codons for a putative leader peptide, ribosome-binding site, terminator sequence and structural gene encoding plantaricin ZJ5 was identified (Figure 4). Although the amino acid sequence of the mature PZJ5 peptide did not show significant homology to any other known bacteriocins, PZJ5 was found to contain a well-known leader peptide characterized by two conserved glycine residues. This leader peptide is found in many bacteriocins. This type of peptide is believed to cleave the N-terminal leader peptide from the bacteriocin to perform at the Gly-Gly site and to transport the resulting mature polypeptide across the plasma membrane [25], [28], [29], [30]. The molecular weight of PZJ5 was calculated to be 2631.1 Da; this value is approximately 57.2 Da higher than the value obtained by mass spectrometry. This result also suggests a post-translational modification in PZJ5, but the nature of the modification is unknown.

In recent years, a variety of LAB bacteriocins have attracted attention due to their potential application as the next generation of antimicrobial compounds used in food preservation [31]. *L. plantarum* is one of the major LABs, and it can be isolated from a variety of fermented vegetables [32], [33]. *L. plantarum* ZJ5 produces the promising novel bacteriocin plantaricin ZJ5, which exhibits activity over a wide range of pH, heat, and bactericidal antimicrobial spectrum while at the same time being safely degradable by human digestive enzymes. Because of these properties, it is considered that plantaricin ZJ5 will be used as a novel biopreservative.

## Supporting Information

Table S1 **Stability of purified Plantaricin ZJ5 subjected to different pH treatments.**
(DOC)
