# Supplementary material for: Purification and Characterization of Plantaricin ZJ5, a New Bacteriocin Produced by Lactobacillus plantarum ZJ5
Source: PLoS One. 2014 Aug 22;9(8):e105549. doi: 10.1371/journal.pone.0105549 (PMC4141769; doi:10.1371/journal.pone.0105549)
Supplement: Table S1 — Stability of purified Plantaricin ZJ5 subjected to different pH treatments. (DOC) [file pone.0105549.s001.doc]

**Table S1.**

Stability of purified Plantaricin ZJ5 subjected to different pH treatments

| pH | Relative activity (%)^a^ |
| --- | --- |
| 2.0 | 100 |
| 3.0 | 100 |
| 4.0 | 100 |
| 5.0 | 100^b^ |
| 6.0 | 100 |
| 7.0 | 30 |
| 8.0 | 30 |

^a^ The PZJ5, in different pH solutions, was incubated for 2 h at 37°C and the pH was reverted to 5.0.

^b^ The activity of an untreated sample (pH 5.0) was defined as 100%.
